# Methylation of *MGMT* and *ADAMTS14* in normal colon mucosa: biomarkers of a field defect for cancerization preferentially targeting elder African-Americans

**DOI:** 10.18632/oncotarget.2852

**Published:** 2015-02-03

**Authors:** Sergio Alonso, Yuichi Dai, Kentaro Yamashita, Shina Horiuchi, Tomoko Dai, Akihiro Matsunaga, Rosa Sánchez-Muñoz, Cristina Bilbao-Sieyro, Juan Carlos Díaz-Chico, Andrei V. Chernov, Alex Y. Strongin, Manuel Perucho

**Affiliations:** ^1^ Tumor Initiation and Maintenance Program, Sanford-Burnham Medical Research Institute (SBMRI), La Jolla, California, USA; ^2^ Institute of Predictive and Personalized Medicine of Cancer (IMPPC), IGTP, Badalona, Barcelona, Spain; ^3^ Dept. of Pathology, Graduate School of Comprehensive Human Sciences, University of Tsukuba, Ibaraki, Japan; ^4^ Dept. of Diagnostic Pathology, Tsukuba Memorial Hospital, Tsukuba, Ibaraki, Japan; ^5^ Dept. of Biochemistry and Molecular Biology, Cancer Research Institute of The Canary Islands, University of Las Palmas de Gran Canaria, Spain; ^6^ Cancer Research Center, Sanford-Burnham Medical Research Institute (SBMRI), La Jolla, California, USA; ^7^ Instituciò Catalana de Recerca i Estudis Avançats (ICREA), Barcelona, Spain

**Keywords:** *KRAS* mutations, *TP53* mutations, *MGMT*, O^6^-methylguanine-DNA methyltransferase, *ADAMTS14*, CRC, colorectal cancer

## Abstract

Somatic hypermethylation of the O^6^-methylguanine-DNA methyltransferase gene (*MGMT*) was previously associated with G > A transition mutations in *KRAS* and *TP53* in colorectal cancer (CRC). We tested the association of *MGMT* methylation with G > A mutations in *KRAS* and *TP53* in 261 CRCs. Sixteen cases, with and without *MGMT* hypermethylation, were further analyzed by exome sequencing. No significant association of *MGMT* methylation with G > A mutations in *KRAS*, *TP53* or in the whole exome was found (*p* > 0.5 in all comparisons). The result was validated by *in silico* comparison with 302 CRCs from The Cancer Genome Atlas (TCGA) consortium dataset. Transcriptional silencing associated with hypermethylation and stratified into monoallelic and biallelic. We also found a significant clustering (*p* = 0.001) of aberrant hypermethylation of *MGMT* and the matrix metalloproteinase gene *ADAMTS14* in normal colonic mucosa of CRC patients. This suggested the existence of an epigenetic field defect for cancerization disrupting the methylation patterns of several *loci*, including *MGMT* or *ADAMTS14*, that may lead to predictive biomarkers for CRC. Methylation of these *loci* in normal mucosa was more frequent in elder (*p* = 0.001) patients, and particularly in African Americans (*p* = 1 × 10^−5^), thus providing a possible mechanistic link between somatic epigenetic alterations and CRC racial disparities in North America.

## INTRODUCTION

The etiology of oncogenic mutations in colorectal cancer (CRC) is only explainable in cancers with microsatellite instability (MSI) [1]. MSI is originated by defects in the DNA mismatch repair (MMR) system and is the hallmark of the Hereditary Non-Polyposis Colon Cancer (HNPCC) syndrome [2, 3]. Germ line mutations in MMR genes, combined with other somatic alterations in the remaining allele impair MMR and, as a result, hundreds of thousands of spontaneous DNA replication errors accumulate in the genome in the course of multiple consecutive cell replications. MSI is also manifested in approximately 10–15% and 15–20% of non-hereditary CRC and endometrial cancer (EC) [1–4], predominantly as a result of epigenetic silencing of *MLH1* linked to promoter hypermethylation [5, 6].

MSI tumors display a mutator phenotype that raises the tumor cell mutation rate two to three orders of magnitude over that of normal cells [2, 7]. Paradoxically, despite their mutator phenotype, the frequency of mutations in the prototypical cancer genes for CRC, *KRAS* and *TP53*, is lower in MSI tumors than in tumors without MSI [1]. This can be explained as the MSI mutator phenotype leads to biallelic mutations in other oncogenic target genes such as *TGFRBII* and *BAX* [8, 9].

Activation of *ras* oncogenes is implicated in human carcinogenesis [10], mainly by promoting cellular proliferation and inhibiting apoptosis [11]. *KRAS* mutations occur frequently in tumors of the pancreas and the lung, and colorectal adenomas and carcinomas [12, 13]. Point mutations of *KRAS* also occur in 10% to 40% of EC [14].

The tumor suppressor gene *TP53* is a checkpoint regulator that has been named the “guardian of the genome” [15]. The frequency of *TP53* somatic point mutations in CRC is estimated to be above 50%. The majority (approximately 80%) are G > A missense transition mutations at CpG dinucleotides that mainly occur in five hotspot codons (175, 245, 248, 273, and 282) [16].

O^6^-methylguanine-DNA methyltransferase (MGMT) is an ubiquitous DNA repair enzyme that removes mutagenic and cytotoxic adducts from the O^6^-guanine in DNA, the preferred point of attack by many carcinogens and alkylating chemotherapeutic agents [17]. Alkylating agents are also provided by N-nitrosation of amines derived from protein catabolism that occurs primarily in the acid environment of the stomach [18]. Thus, lack of MGMT function has a mutagenic effect that leads to G to A transition mutations. The human *MGMT* gene has a normally unmethylated promoter CpG island that, when hypermethylated, correlates with transcriptional silencing [19, 20].

A causal relationship between *MGMT* silencing and somatic mutations in *KRAS* and *TP53* in CRC was initially proposed based on the association between *MGMT* hypermethylation and G > A transitions in *KRAS* and *TP53* [21, 22]. This view remains widely accepted [23–25], despite some reports with contradictory results [26–29].

SNS can be transition or transversion mutations. Transitions change one purine for another purine (G→A or A→G), or pyrimidine for another pyrimidine (C→T or T→C). G→A transition is the same as C→T transition (G→A in one strand and C→T in the other). For simplification we refer it as G > A transition. Most SNS at codons 12 (GGT) and 13 (GGC) of *KRAS*, activate its oncogenic activity. The most frequent *KRAS* mutations in CRC are G > A transitions at the second G of these triplets, leading to a glycine→aspartic acid substitution: GGT→GAT or GGC→GAC. In *TP53*, SNS are spread over the gene coding region. Of the G > A transitions, those occurring in CpG dinucleotides CG → TG (GC →AC in the reverse strand) were the most abundant in *TP53* (81%).

In this study, we analyzed the association between *KRAS* and *TP53* missense mutations with *MGMT* methylation in CRC with and without MSI. The relationship between these genetic and epigenetic somatic alterations was also expanded by exome sequencing and by *in silico* analysis of the publicly available data from The Cancer Genome Atlas (TCGA) consortium, (http://cancergenome.nih.gov/). As MSI CRC is very different from CRC without MSI (MSS) in genotype and phenotype, the inclusion of MSI in CRC analysis is a confounding factor. We then focused on the analysis of MSS cancers for the studies on methylation and mutation.

We also analyzed the methylation of *ADAMTS14,* a member of the ADAM/ADAMTS gene family that plays a role in cell migration and invasion, recently found altered genetically and epigenetically in carcinogenesis in general and CRC in particular [30–33]. We explored the relationship between *ADAMTS14* and *MGMT* methylation in normal and tumor tissues of CRC patients and correlated these findings with tumor genotype and phenotype, including clinico-histological parameters.

## RESULTS

### *MGMT* methylation, MSI, *KRAS* and *TP53* mutations in CRC

**Figure 1:**
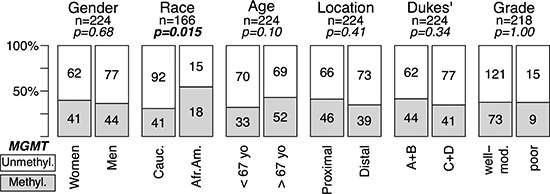
*MGMT* methylation and genotype/phenotype of CRC without MSI Data for MSI positive tumors are in Supplementary Figure S4. *MGMT*-hypermethylated tumors, grey; tumors without hypermethylation, white. Gender, woman vs. men; Race, Caucasian (Cauc.) vs. African American (Afr.Am.); Age, below median (<67 years) vs. above median (>67 years); Location, Proximal (proxim.) includes cecum, ascending and transversal colon vs. Distal, descending, sigmoid and rectum; Invasiveness, Dukes' A+B vs. Dukes' C+D; Differentiation, moderate to well differentiated (Mod-Well) vs. poorly differentiated (Poor). *MGMT* methylation was assessed by MSP (see Materials and Methods). *p*-values were calculated by univariate Fisher's tests. In bold, statistically significant values.

In the complete series of 735 CRC 89 (12.1%) were classified as MSI, and 432 were analyzed for single base substitution (SNS) mutations in *KRAS* and 392 for SNS mutations in *TP53*. A subset of 261 cases was also analyzed for *MGMT* methylation. The associations between MSI, *KRAS* and *TP53* mutations with CRC genotype (oncogenic mutations) and phenotype (clinico-pathological features) are summarized in supporting Figures S1–S3. *MGMT* methylation associated with African Americans (*p* = 0.015) and there was a trend for older patients (*p* < 0.1) in CRCs without MSI (Figure 1). No association was found between methylation and location, stage and grade in tumors without (Figure 1) or with (Figure S4) MSI. All subsequent results describe tumors without MSI unless otherwise specified.

### *MGMT* methylation and type of *KRAS* and *TP53* mutations

**Figure 2:**
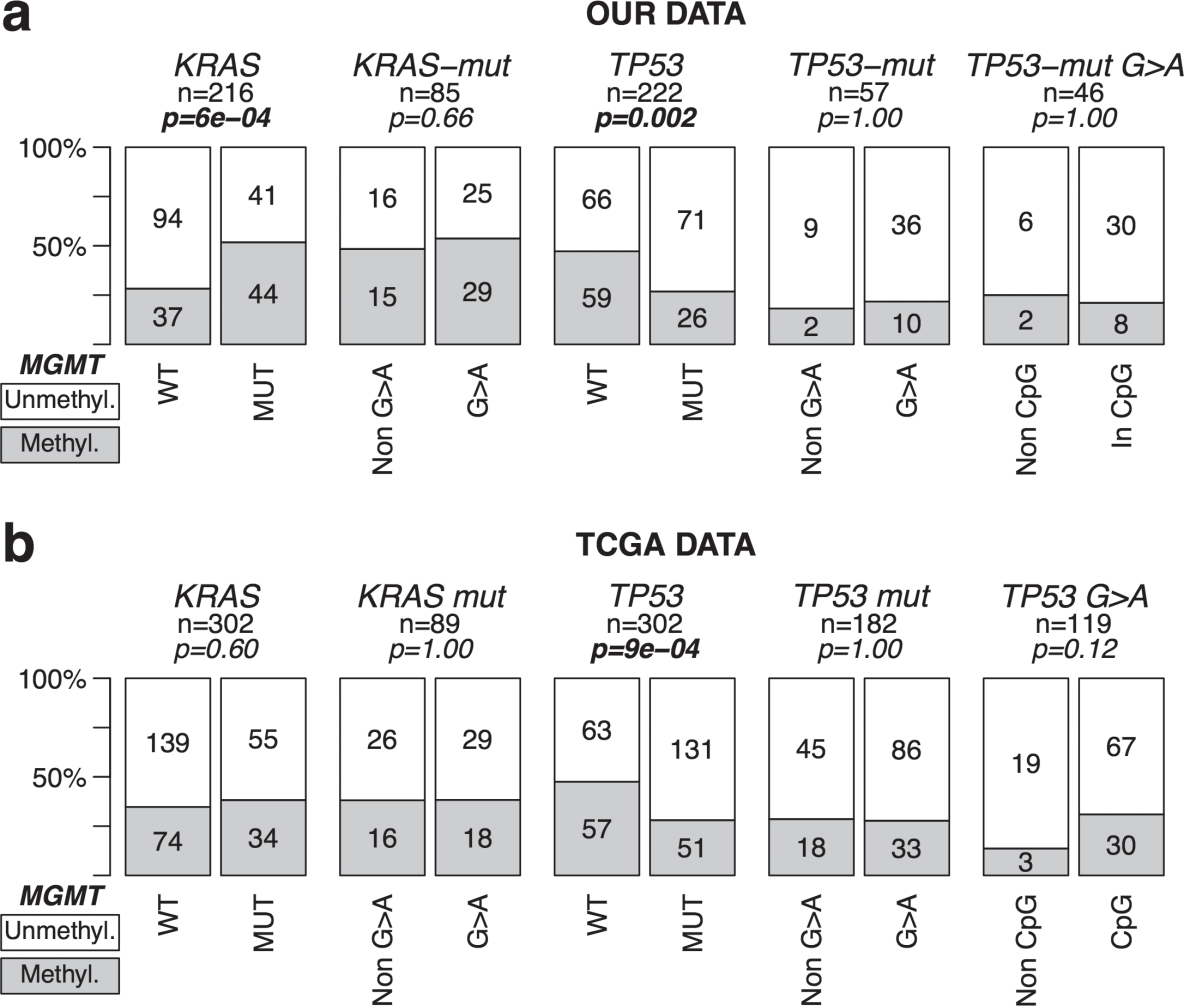
*MGMT* methylation and mutations in *KRAS* & *TP53* in CRC without MSI Symbols and codes as in Figure 1. WT; wild type; MUT, mutant. In the upper row, data from our tumor collection. Mutations refer to SNS at codons 12 and 13 of *KRAS* and in exons 4–9 of *TP53*. In the lower row, TCGA COAD + READ datasets, excluding tumors classified as hypermutated (MMR defective, MSI). Only non-silent SNS mutations occurring in the whole coding sequence of *TP53*, and in codons 12 and 13 of *KRAS* were considered for the analysis.

In CRC, *MGMT* methylation associated positively with *KRAS* mutations (28% vs. 52% *p* = 0.0006) and negatively with *TP53* mutations (47% vs 27%, *p* = 0.002) (Figure 2 top). *MGMT* methylation had no significant association with G > A transitions in *KRAS* (48% vs. 54%, *p* = 0.66) and *TP53* (18% vs. 22% *p* = 1.0) (Figure 2a). There was no association between *MGMT* methylation and *TP53* G > A mutations in CpG sites (Figure 2a).

Multivariate logistic regression analysis including all the genotypic and phenotypic characteristics of CRCs (Figures 1 and 2a), confirmed the statistically significant association of *MGMT* methylation with African Americans (OR = 3.11, *p* = 0.013) and *KRAS* mutations (OR = 2.62, *p* = 0.013), and the negative association with *TP53* mutations (OR = 0.41, *p* = 0.022) (Figure S5).

To further assess our findings, we analyzed the matching normal and tumor samples from 36 CRCs, using Illumina HM450K arrays. Samples were classified into *MGMT*-methylated or demethylated based on the values of three probes located within the *MGMT* 5′ CpG island and enhancer region that exhibits a bimodal distribution in tumor samples (Materials and Methods, Figures S6 and S7). The concordance of the Illumina HM450K results with the previous MSP-based classification was 93%. We therefore used these three probes to explore the available public data from the Cancer Genome Atlas (TCGA) consortium on CRC (COAD plus READ databases, Figure 2b) [34]. There was no correlation between *MGMT* methylation and *KRAS* SNS in codons 12 and 13 (*p* = 0.60), and non-silent *TP53* SNS were fewer in tumors with *MGMT* methylation (*p* = 9 × 10^−4^). There was no significant difference in the relative frequency of G > A transitions in tumors with and without *MGMT* methylation in either gene.

### *MGMT* methylation and exome mutation spectrum

To study the relationship between *MGMT* hypermethylation and the overall incidence of somatic G > A transitions, we performed exome sequencing in 18 CRCs and their matched normal tissues, 9 of them with *MGMT* hypermethylation. To reduce confounding factors, we selected only proximal colon cancers without MSI from Caucasian patients.

**Figure 3:**
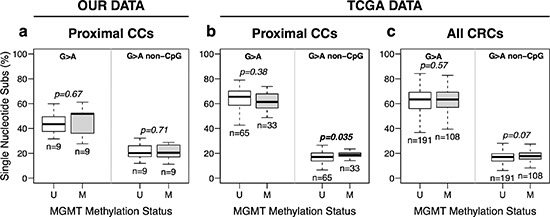
*MGMT* methylation and somatic G > A transition mutations in CRC without MSI **(a)** Data from exome sequencing of eighteen pairs of matching Normal-Tumor samples, 9 with (M) and without (U) *MGMT* hypermethylation, from our tumor collection. **(b** and **c)** data from proximal (b) and all (c) CRCs cancers from the TCGA, excluding hypermutated (MSI) tumors. Box plots represent the median percent of G > A transition mutations vs. all other single base substitutions. *p*-values were calculated by Student's *t*-test.

The exome sequencing data was used to estimate the mutation spectrum in cancers with and without *MGMT* methylation. Figure 3a shows that there was no difference in the frequency of G > A transitions regardless of *MGMT* methylation status. Similar result was derived in our *in silico* analysis of the TCGA consortium data. Only a weak positive association between *MGMT* methylation and G > A transitions in non-CpG sites was detected for both the entire series of cancers including the rectum (*p* = 0.07) and proximal cancers (*p* = 0.035) (Figure 3b and 3c). Multivariate regression analysis also revealed that *MGMT* methylation had no detectable effect in the frequency of SNS, including G > A transitions, in CRC without MSI (Figure S9). Instead, patient age showed a significant association with mutation for all SNS (*p* = 0.009) or G > A transitions (*p* = 1.2 × 10^−4^), especially those taking place in CpG sites (*p* = 1.0 × 10^−5^), but not for non-G > A SNS (*p* = 0.665) or G > A transitions in non CpG sites (*p* = 0.131) (Figure S9).

### *MGMT* methylation and mRNA expression

**Figure 4:**
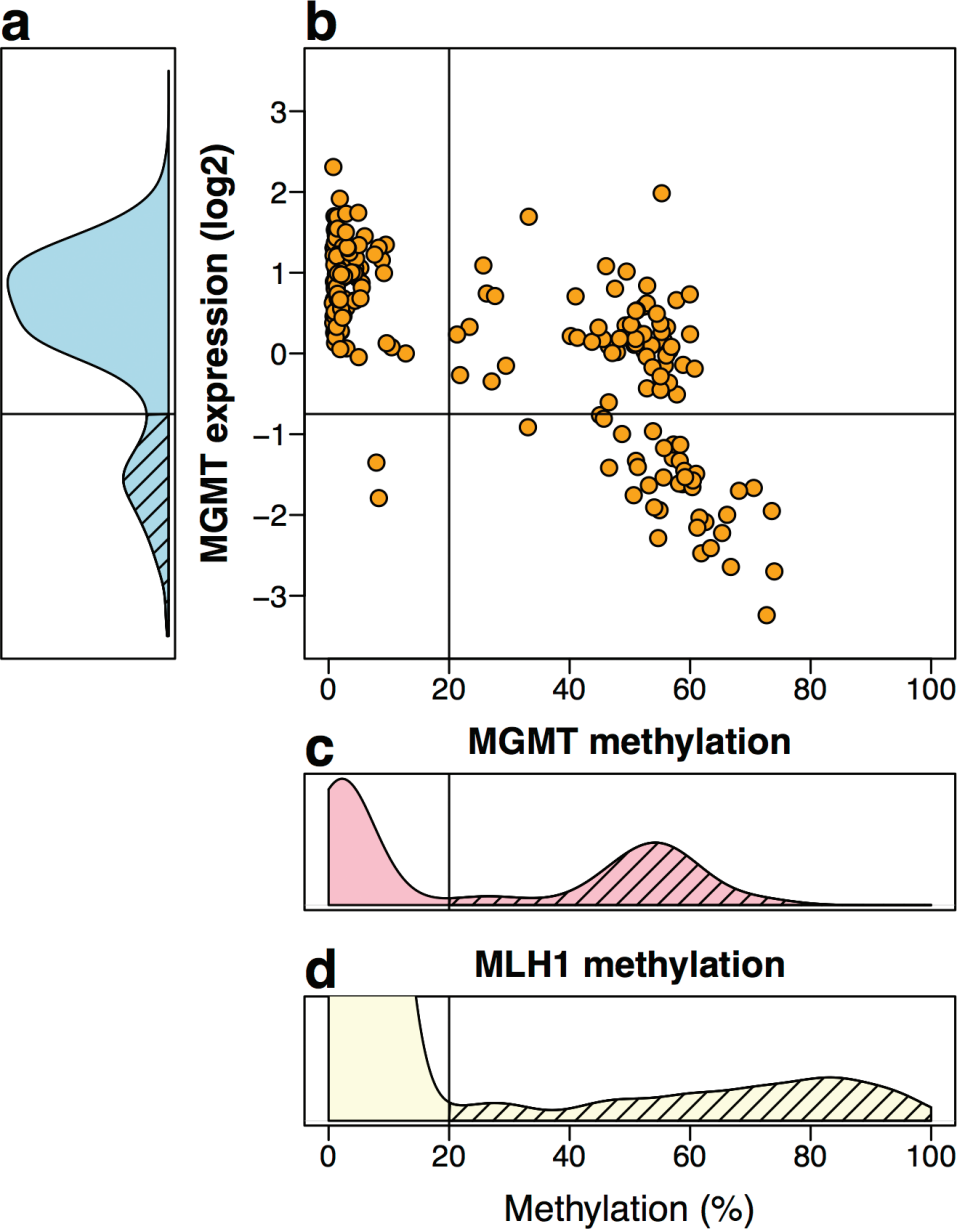
*MGMT* methylation and expression *in silico* TCGA data of 222 CRC **(a)** Distribution of *MGMT* expression levels by Agilent two-color expression microarrays, and normalized relative to the average expression level of 22 normal samples. Yellow, orange and red lines indicate the mean expression values of the groups defined in b. **(b)** Dot-plot of methylation levels vs. expression levels. By cutting at a relative expression of 0.36, and methylation level of 20% (ß-value = 0.2), three groups of tumors are defined: 54% of tumors with no *MGMT* methylation and average expression level similar to that of the normal tissues (yellow circles), 27.5% of tumors with methylation and a moderate reduction in expression (orange squares), 18.5% of tumors with reduction in expression, most but not all with methylation (red diamonds). **(c)** Distribution of the average methylation of probes cg12434587, cg12981137 and cg02941816, located in the promoter region of *MGMT*. In the normal tissues available at the TCGA, these probes never showed methylation above 20%. Of the 222 cases analyzed, 45.05% exhibited an average methylation above 20% (dashed area) with a peak centered around 50%. Association of *MGMT* methylation with lower expression was very significant (*r* = −0.67, 95%CI = [−0.73,−0.59], *p* < 2.2 × 10^−16^, by Pearson's product-moment correlation test). **(d)** Methylation status of *hMLH1* promoter (probe cg13846866) is shown for comparison from a comprehensive larger dataset of CRC from the TCGA. About 12% of the tumor samples (62/514) exhibited methylation above 20% (dashed area), with a peak centered around 80%.

*MGMT* methylation assessed by Illumina microarrays in the TCGA CRC samples showed a clear bimodal distribution (Figures 4c and S6). We classified samples into *MGMT* methylated or *MGMT* unmethylated by setting the ß-value threshold of 0.2, at the valley of the bimodal distribution of methylation. Notably, the second peak of this distribution was centered at a ß-value 0.5, suggestive of monoallelic methylation (Figure 4c and S6). Similar results were derived from our sample collection (Figure S7). In contrast, the methylation distribution of the *MLH1* gene, which is known to undergo transcriptional silencing by promoter methylation in CRC with MSI, showed a methylation peak above ß-value of 0.8, indicating biallelic methylation or monoallelic methylation accompanied by loss of the unmethylated allele (Figure 4d). *MGMT* expression of the TCGA CRC samples (as assessed by Agilent expression microarrays), also exhibited a bimodal distribution, with 81.3% of the samples with an expression level above 0.36 (relative to the average expression of *MGMT* in 22 normal colon samples, Figure 4a), used as cut-off for *MGMT*-silencing because it coincided with the valley separating the two expression peaks.

*MGMT* methylation associated with lower expression (*r* = −0.67, *p* < 2.2 × 10^−16^, Figure 4b). However, in the majority of *MGMT* methylated cases mRNA levels were roughly equal to 50% of the levels in the negative cases, again suggesting that epigenetic silencing affected only one allele (Figure 4b). A minority of *MGMT* methylated cases exhibited lower expression, despite their methylation being also around ß = 0.5 (Figure 4b), suggesting that other mechanism, in addition to promoter methylation, was required for full *MGMT* transcriptional silencing in these tumors.

### *MGMT* and *ADAMTS14* methylation in normal mucosa of CRC

**Figure 5:**
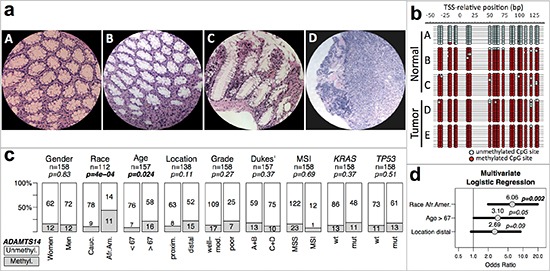
*ADAMTS14* methylation and genotype/phenotype of CRC without MSI **(a)** Micrographs at 200X of hematoxylin-eosin stained representative sections of normal colonic tissue from a patient without (A) and a patient with (B and C) *ADAMTS14* hypermethylation, and tumor tissue from the latter patient (D) **(b)** Bisulfite sequencing profiles of the *ADAMTS14* 5′ region from –50 to 130 bp, relative to the transcriptional start site (TSS) from DNA isolated from normal and tumor microdissected areas equivalent to the areas in micrographs A–D, and another tumor area from the same patient (E) Red and blue circles represent methylated and unmethylated CpG sites, respectively. **(c)** Associations of *ADAMTS14* hypermethylation in normal mucosa of CRC patients with their phenotype/genotype. Symbols and codes as in Figure 1. **(d)** Forest plot of multivariate logistic regression analysis of the association of *ADAMTS14* hypermethylation with race, age and location. The horizontal bars represent the 95% confidence intervals with the centered circles representing the estimated odds ratio, with values indicated above. *p*-values of the multivariate analysis are also indicated for every parameter included in the model.

*MGMT* has also been found methylated in normal colon mucosa, having been associated with an epigenetic field defect for cancerization in CRC [24, 35, 36]. Albeit reduced compared with the tumors, there was a significant level of methylation in normal mucosa from 7% (11/157) of CRC. In a parallel study we also identified significant methylation of *ADAMTS14* in normal colon mucosa in 14.6% (23/157) of CRC patients (Figure 5a and 5b). *ADAMTS14* methylation in normal mucosa was significantly associated with old age and was more frequent among African-Americans (Figure 5c). These associations remained statistically significant in multivariate logistic regression analysis (Figure 5d), with race being the most significant.

**Figure 6:**
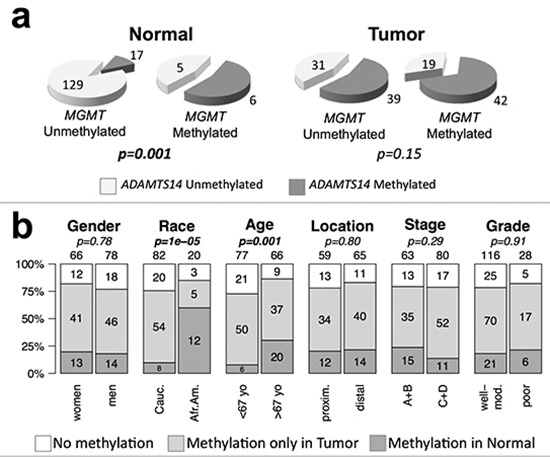
*ADAMST14* and *MGMT* methylation and genotype/phenotype of CRC without MSI **(a)** Clustering of *MGMT* and *ADAMTS14* hypermethylation in the normal mucosa (left) and tumor tissue (right) of CRC patients. *p*-values were calculated by Fisher's exact test. **(b)** Patients were divided into three groups according to the methylation status of *ADAMTS14* and *MGMT*: without methylation in either gene (white), with methylation in one or both genes in the tumor sample but not in the non-tumoral mucosa (light grey), or with methylation of at least one of these genes in the non-tumoral mucosa (dark grey). Symbols and codes as in Figure 1. *p*-values were calculated by χ^2^ test.

There was a significant clustering of methylation of *MGMT* and *ADAMTS14* in normal tissue (Figure 6a). We then reclassified the 144 CRCs without MSI for which complete methylation information for the two genes was available into three groups: i) no methylation in either gene, ii) methylation in one or both genes in tumor, but not in normal tissue, and iii) methylation in normal tissue in at least one of these two genes. Methylation in normal tissue occurred preferentially in elder CRC patients (*p* = 0.001) and African Americans (*p* = 1 × 10^−5^) with CRC (Figure 6b).

## DISCUSSION

Accumulation of mutations in *KRAS* and *TP53* cancer genes is critical for CRC pathogenesis. *MGMT* hypermethylation was reported to associate with *KRAS* and *TP53* G > A transitions, the most frequent single base substitutions in both genes [21, 22]. Thus, critical oncogenic mutations for CRC could be explained by the previous *MGMT* epigenetic silencing. This report shows the absence of association between *MGMT* methylation and G > A transition mutations in *KRAS* and *TP53* genes in CRC without MSI. Because this conclusion was validated by the massive TCGA consortium data, the results do not appear to be due to tumor sample variation.

Next, we performed exome sequencing to determine the somatic mutational spectra of 18 CRC with and without *MGMT* methylation. Again, no association was found between methylation and the proportion of G > A somatic transitions. Identical conclusion was reached by *in silico* analysis of the TCGA consortium: there was no association between *MGMT* methylation and G > A transition mutations in *KRAS* and *TP53* or in the whole exome of 386 CRC without MSI. Therefore, epigenetic silencing of *MGMT* cannot explain the origin of the majority of oncogenic mutations in *KRAS* or *TP53*, in CRC without MSI. In support of this conclusion are our unpublished results with endometrial cancer (EC). Although *KRAS* mutations were identified in 34.1% MSI and 9.2% non-MSI EC (*p* < 0.0002), none of the 204 EC samples exhibited *MGMT* methylation (data not shown).

While in MSI CRC missense mutations in *KRAS* and *TP53* (and in any gene) can be accounted for by the strong mutator phenotype displayed by these tumors, in CRC without MSI the origin of these oncogenic mutations remains unclear [14]. While the spontaneous mutation rate may account for mutations in several cancer genes in stem cells [37, 38], it is very unlikely that the *same* gene accumulates two mutations. Thus, *TP53* somatic biallelic mutations in cancer cannot be explained by the spontaneous mutation rate of normal cells [39]. This also applies to all tumor suppressors and mutators as the MMR genes themselves. *Ras*, however, are oncogenes and in principle a mono-allelic mutation may be sufficient for their activation. Nevertheless, the common occurrence in CRC is that several copies of the mutated allele coexist in tumors with the wild type allele, indicating that the gene acts in a dominant, but dose dependent manner [40–42].

As biallelic missense mutations are virtually absent in CRC without MSI, a plausible hypothesis is that the SNS common for both *KRAS* and *TP53* occur spontaneously (or induced by uncharacterized mutagens) and that subsequent mutations in the other allele typically involve another type of alteration, the occurrence of which is enhanced over the spontaneous rate. Deletions and amplifications of chromosomal segments are the most common, although not unique, mutational events that affect the gene dosage of *KRAS* and *TP53* in CRC. It is uncertain whether these alterations are the consequence of a defect involved in the active generation of chromosomal instability, or just the result of a stepwise augmentation of the probability of incurring into a mitotic error during clonal selection. In the case of *KRAS*, this may be simply due to the increased mitotic activity presumably caused by the mono-allelic mutation. Whether some *TP53* monoallelic missense mutations may also work in a haplo-insufficiency manner increasing the chances of subsequent loss of heterozygosity (LOH) events is also a possibility, especially since the heterogeneity of these mutations [16]. Alternatively, the second event may be influenced by other independent events already present in the tumor cell at that stage of tumor progression.

The weak association of G > A mutations occurring in non-CpG sites in the cancer cell exome (Figures 3 and S9) shows that *MGMT* deficiency may indeed contribute to the accumulation of some of these mutations. However, the difference in mutation frequency is very small, implying that many of the mutations have been already generated prior to the epigenetic silencing of the *MGMT* gene. In fact, the principal SNS in CRC are G > A transitions in CpG sites that accumulate in an age dependent manner (Figure S9), which is consistent with the hypothesis that most SNS take place throughout the entire life of the patient and prior to tumorigenesis, mainly by spontaneous deamination of methylated cytosines [43, 44]. Thus, mutations occurring after *MGMT* silencing may be a minority compared with the previous mutations accumulated during the life of the tumor precursor cells. The vast majority of these “passenger” mutations do not contribute to tumorigenesis, but they are exposed by the clonal expansion of the carrying precursor cells.

Further analysis of exome deep sequencing data may shed light on this issue. Non-clonal mutations (after transformation) would be distinguishable from clonal mutations by the frequency recorded in their sequence reads. The hypothesis can be thus tested by a comparative analysis in tumors with and without *MGMT* methylation of the high and low relative frequency of sequencing reads of G > A vs. non-G > A SNS, and between G > A in CpG vs. non-CpG sites. For instance, in tumors with *MGMT* methylation, the frequency of non-clonal G > A transitions (with low relative number of mutant vs. wild type sequencing reads) in non CpG sequences, would be higher than that from tumors without methylation.

The lack of significant correlation between methylation and mutation also suggests that there might be no significant contribution by alkylating agents in the large intestine to generate these mutations. However, even if the mutator role of *MGMT* hypermethylation appears modest, it may have a relevant prognostic value. Recent clinical studies showed that methylation of *MGMT* is a useful predictor of the responsiveness of tumors to alkylating agents in gliomas [45, 46], and is associated with good survival in patients treated with multidrug regimens [47]. It also predicts the response to the alkylating drug dacarbazine in metastatic CRC [48, 46]. In this context, the data on *MGMT* methylation and expression reveals a complex pattern of regulation and argues against a complete silencing in a significant proportion of methylated cases. This may have potential impact on current treatment regimens of CRC. We found that methylation of *MGMT* seems to be predominantly monoallelic in CRCs without MSI, with just a 50% reduction in transcriptional levels in the majority of the cases. It would be interesting to evaluate in future studies the response to these alkylating drugs in patients stratified in three groups (Figure 4), distinguishing between no silencing and mono-allelic or biallelic silencing.

In contrast with the clear asymmetries observed for MSI, *KRAS* and *TP53* mutations, the only significant association found for *MGMT* methylation was with African-American CRC patients that tended to be older (Figure 1 and Figures S1–S3). In an independent study, we also observed a significant association of hypermethylation of *ADAMTS14* in the normal colon mucosa mainly in elder African-Americans with CRC (Figure 5). The tendency of *ADAMTS14* hypermethylation in normal mucosa to occur in the distal colon distinguishes this phenomenon from the generalized hypermethylation that is more prominent in tumors of the proximal colon [49–51]. Clustering of methylation of *MGMT* and *ADAMTS14* (Figure 6a) in normal mucosa supports the concept of a field for cancerization in some individuals at high risk for CRC that involves the aberrant methylation of several *loci*, including, but not restricted to *MGMT* and *ADAMTS14* [24, 36]. We do not suggest, however, that these genes play an active role in this field defect. Probably they are just markers of a more generalized epigenetic dysfunction that underlies an apparent high risk for CRC, preferentially among elderly African-Americans (Figure 6b).

We cannot distinguish at this point between ethnic or environmental causes for this association, and this issue remains the subject for further studies. We also do not know whether this phenomenon is restricted to those individuals already with CRC or if the alteration occurs, as we suspect, in pre-symptomatic individuals. The geography of this field defect also needs to be surveyed to discriminate between systemic or focalized defect. This in turn may have practical consequences for diagnosis of the predictive lesions. Although their detection may be a challenging task, epigenetic alterations can be detected in liquid biopsies. In conclusion, our findings provide mechanistic clues for the ethnic disparities known to affect colon cancer [52], and a rationale for novel CRC predictive tests.

## PATIENTS AND METHODS

### Study participants and samples

Unselected primary CRC and corresponding non-tumoral tissues were obtained through the Cooperative Human Tissue Network (CHTN) from 751 patients who underwent curative surgery in various hospitals of Philadelphia, Tennessee, Ohio and Alabama between 1985 and 2004. Only patients with adenocarcinomas were included in the study. Clinical information included age at diagnosis, gender, race, tumor location, and surgical stage; and pathology data included grade of differentiation. Tumor staging was based on Duke's classification. Sanford-Burnham Institutional Review Board approval was obtained for this work. We also retrospectively studied 204 consecutive patients with EC (FIGO stage I-III), diagnosed and treated at the Department of Obstetrics and Gynecology of the Gran Canaria's Hospital Universitario Materno Infantil (Canary Islands, Spain) between 1990 and 1999. The ethics committees from Gran Canaria's Hospital Universitario Materno Infantil approved the research protocol, which was in compliance with national legislation and performed according to the ethical guidelines of the Declaration of Helsinki [53]. After surgical resection, samples were rapidly frozen in liquid nitrogen and stored at −80ºC until further use. DNA was isolated by standard procedures involving mechanical disruption of tissue, SDS lysis and proteinase K digestion, phenol-chloroform extraction, and ethanol precipitation [54].

### MSI analysis, *KRAS* and *TP53* mutation detection

MSI was determined in 751 CRC as previously described. [55] Codons 12/13 of the *KRAS* oncogene in 432 CRC, and exons 4 to 8 of the *TP53* suppressor gene in 392 CRC were analyzed by single stranded conformation polymorphism (SSCP) and DNA sequencing. PCR primers and conditions for the amplification and analysis of these genes are detailed in supporting materials SM1.

### Methylation analysis of *MGMT* and *ADAMTS14*

The methylation status of *MGMT* was determined in 273 CRC samples by methylation-specific single PCR (MSP) [56] of a specific sequence region where methylation invariably correlates with lack of *MGMT* expression [19, 20]. PCR primers and conditions are described in supporting materials SM2. The promoter region of *ADAMTS14* was analyzed by bisulfite sequencing and combined bisulfite and restriction analysis (COBRA) in 158 CRC patients (supporting materials SM3) [57, 58].

### Exome sequencing

We sequenced 36 exomes corresponding to tumor tissue and corresponding normal mucosa from 18 patients with proximal colon cancer (9 with and 9 without *MGMT* methylation), using Illumina TruSeq Enrichment protocol and HiScan-SQ platform. Somatic mutations were determined by comparing the tumor and normal exome from the same individual. Detailed information about the library preparation, sequencing protocol and bioinformatic tools is provided in supporting information SM4. After exome sequencing, over 100 Single nucleotide variants (SNVs) and somatic mutations were manually selected for validation by PCR and sequencing (supporting materials SM5).

### *In silico* analysis of TCGA data

We combined the data from the COAD (colon adenocarcinoma) and READ (rectum adenocarcinoma) databases from the TCGA (https://tcga-data.nci.nih.gov/) [34]. In total, we obtained clinical information from 592 patients, methylation data from 583 primary CRCs, and exome mutational data from 386 CRCs (supporting dataset S1). *MGMT* methylation status was determined by Illumina HM27/HM450 probes cg12434587, cg12981137 and cg02941816, that interrogate three CpG sites located –239 bp, 128 bp and 248 bp from the *MGMT* transcriptional start site, respectively, within regions that associate with transcription of *MGMT*. [20] A detailed explanation of the TCGA datasets we employed in this work and the classification criteria for MSI and *MGMT* status are provided in supporting materials SM6.

### Statistical analysis

Statistical analyses were performed using R environment [59]. Associations in categorical data were analyzed by two-sided Fisher's exact tests (for 2 × 2 contingency tables) or Chi-squared test with Yates' correction (for larger contingency tables). Multivariate analyses were performed by logistic regression, with stepwise reduction of non-significant factors and interactions. Forest plots represent the odds ratios (OR) and the 95% confidence intervals for each parameter included in the model. In box plots, the boxes represent the interquantile range (IQR) with a horizontal bar indicating the median. Dispersion bars indicate the highest and lowest datum within 1.5 × IQR of the lower quartile and 1.5 × IQR of the highest quartile. Statistical significance threshold was set at *p* ≤ 0.05.

## SUPPLEMENTARY FIGURES AND TABLE


