# Supplementary material for: Methylation of MGMT and ADAMTS14 in normal colon mucosa: biomarkers of a field defect for cancerization preferentially targeting elder African-Americans
Source: Oncotarget. 2015 Feb 3;6(5):3420–31. doi: 10.18632/oncotarget.2852 (PMC4413663; doi:10.18632/oncotarget.2852)
Supplement: Supplementary file 1 [file oncotarget-06-3420-s001.pdf]

EXPERIMENTAL PROCEDURES

SM1. detection of *KRAS* and *TP53* mutations

For *KRAS* analysis, 20 ng of genomic DNA were amplified by PCR using primers 5′-TAAGGCCTGCTGAAAATGA-3′ (forward) and 5′ GTCCTGCACCAGTAATATGC-3′ (reversal). PCR cycling was 94ºC for five minutes, followed by 30 cycles at 94ºC for 30 seconds, 55ºC for 30 seconds, and 72ºC for 90 seconds. A final extension step at 72ºC for seven minutes was performed. For *TP53* analysis, PCR conditions were identical than those for *KRAS* amplification, except for the T_m that was optimized for every PCR. Primers flanking the exons of interest are listed in supporting table S1.

Single stranded conformation polymorphism (SSCP) analysis was used to screen for mutations within the codon 12/13 region of the *KRAS* gene and *TP53* gene [1]. Five µl of PCR product was denatured for 5 minutes at 96ºC with 50 µl of formamide denaturing dye mixture (94% formamide, 10 mM EDTA, 0.3% xylene cyanol, and 0.3% bromophenol blue) and quickly cooled on ice. Amplified products were resolved by electrophoresis in 8% polyacrilamide gels, then stained with ethidium bromide and visualized on a UV transilluminator.

SM2. *MGMT* promoter methylation analysis

MSP

One µg aliquots of the tumor and normal DNA specimens were treated with bisulfute [2, 3] and subjected to PCR with primers 5′-TTGTGTTTTGATGTTTGTAGGTTTTTGT-3′(forward) and 5′-AACTCCACACTCTTCCAAAAACAAAACA-3′ (reverse) for amplification of unmethylated DNA and 5′-TTTCGACGTTCGTAGGTTTTCGC-3′ (forward) and 5′- GCACTCTTCCGAAAACGAAACG-3′ (reverse) for amplification of methylated DNA [4]. PCR cycling conditions were 95ºC for 5 minutes, followed by 35 cycles of 95ºC for 30 seconds, 60ºC for 30 seconds and 72ºC for 30 seconds, and finally, 72ºC for 4 minutes. Each PCR assay included positive and negative controls (DNA from SW48 and LoVo cells, respectively) [5]. PCR products were resolved on 2% agarose gels, stained with ethidium bromide and visualized under UV illumination. Samples with detectable methylation by MSP were considered positive for *MGMT* promoter methylation (Figure S5).

Illumina human methylation arrays 450K

One µg of DNA was treated with . Befor treatment, DNA concentration, integrity and purity were determined by Qubit DNA High Sensitivity fluorimetry, agarose gel electrophoresis and nanodrop, respectively. The efficiency of the bisulfite treatment was tested by PCR amplification of ADAMTS14 promoter region (see SM3). Transformed DNA was subjected to hybridization on Illumina Human Methylation Arrays 450K following manufacturer's protocol. Results were analyzed using RnBeads software [6].

SM3. methylation analysis of *ADAMTS14*

One µg of genomic DNA was treated with EZ DNA Methylation™ Kit (Zymo Research, CA). After treatment, DNA was subjected to PCR amplification with primers 5′- GTTTTTAGTTTGGGATTTGG-3′ and 5′-AACAACCTTAAACCACCCTAAC-3′ that amplify a 214 bp sequence containing 24 CpG sites at the 5′ end of *ADAMTS14*. PCR reactions were performed with Qiagen HotStart Kit, in 25 µl of 1X Buffer, 0.5X Q-Solution, 0.125 mM dNTPs, 0.4 µM of each primer, 100 ng of bisulfite-treated genomic DNA and 1 unit of polymerase. Cycling conditions were as follows: 15 min at 95ºC, followed by 35 cycles of 30 sec at 95ºC, 30 sec at 55ºC and 30 sec at 72ºC, and a final extension of 10 min at 72ºC. For bisulfite sequencing, PCR amplification products were cloned into pCDNA3.2 TOPO-TA (Qiagen) and transformed into *E. coli*. Plasmids from individual clones were isolated and sequenced with primer T7. For COBRA, PCR products were digested with *Bst*UI for 2 h at 60ºC, resolved by electrophoresis in 12% acrylamide gels and visualized on a UV transilluminator after staining with ethidium bromide.

SM4. exome DNA library preparation, sequencing and analysis

DNA concentration, integrity and purity were determined by Qubit DNA High Sensitivity fluorimetry, agarose gel electrophoresis and nanodrop, respectively. 2.6 µg of high-quality genomic DNA in 130 µl of low TE (10 mM Tris pH 7.5, 0.1 mM EDTA pH 8) were fragmented with the Covaris S2 system in 8 stages of 30 seconds. Settings were: duty cycle = 10%, intensity = 5, bursts per second = 200, duration = 240 seconds, mode = frequency sweeping, power = 23 W, temperature 5.5–6.2ºC. Fragmentation size was assessed on 1 µl of 1:10 dilution in water on a High Sensitivity DNA chip assay on the Bioanalyzer 2100 (Agilent) for an average median target size of 200–300 bp and well within the 100–900 bp range recommended by Illumina. 1 µg of fragmented DNA was used as input for the TruSeq DNA library protocol (Illumina) and subject to end repair (30 min at 30ºC), A-tailing (30 min at 37ºC) and indexed adapter ligation (10 min at 30ºC), with intermediate AMPure XP magnetic bead cleanup steps, followed by PCR amplification on a BioRad Dyad thermocycler (denaturation: 30 sec 90ºC; cycling 10 times: 10 sec 90ºC, 30 sec 60ºC, 30 sec 72ºC; extension: 5 min 72ºC). 500 ng of each of 6 libraries with different

indices were pooled and subjected to two rounds of targeted hybridization overnight at 58ºC, with subsequent washing and elution of the captured sequences, followed by a final PCR library amplification step of 10 additional cycles, as above (TruSeq Enrichment, Illumina). Pooled exome libraries were quantified using the KAPA SYBR Fast qPCR kit for Illumina and their size profile was assessed on a High Sensitivity DNA chip on the Bioanalyzer 2100. Exonic sequence fold-enrichment efficiency was verified by qPCR using an exon specific (HTT_F 5′ CCTCCCACATGTCATCAGC 3′ and HTT_R 5′ GCAACCACCTCAAGCACAG 3′) and an intergenic specific primers set (BETA-ACTIN-LEFT 5′ AGTGTGGTCCTGCGACTTCTAAG3′; BETA -ACTIN-RIGHT5′ CCTGGGCTTGAGAGGTAGAGTGT 3′), with 400–1000 fold enrichment in all cases. Each exome library pool was clustered on three lanes of a paired end flow cell in a cBot (Illumina) at 10–12 pM and sequenced using SBS v3 reagents (2 × 101 bp) on a HiScan-SQ system (Illumina), to achieve an average of 2 exomes per lane. Raw sequence data was monitored using the Real Time Analysis software from Illumina to assess cluster density and base quality during the run. It was subsequently exported offline to a computing cluster and storage facility. Basecalling was performed with CASAVA 1.8.2 from Illumina. Data was processed using an in house pipeline that involves the following steps: first, reads are trimmed using trimmomatic [7] and aligned using BWA [8], followed by exome capture on target rate and uniformity assessment using the TEQC Bioconductor package [9]. After alignment, duplicates were marked using Picard (http://picard.sourceforge.net) and alignments around indel regions were refined by local realignment followed by Base Quality Recalibration algorithm with GATK [10]. Variant calling was performed using the UnifiedGenotyper from GATK followed by the application of the Variant Quality Score Recalibration (VQSR) algorithm by GATK [11] in independent steps for SNP or indel calling. Finally, SNPs and indel variants were merged and filtered using GATK and annotated using the SNPeff tool [12]. We performed an additional quality control step by determining the concordance of exonic SNP between the sequencing and Illumina Exon v 1.0 or v 1.1 bead arrays finding it higher than 95% for heterozygous SNPs and between 97 and 98% for homozygous SNPs in all cases. Germline variants and somatic mutations were identified using VarScan [13] with default parameters. Only somatic mutations with a frequency over 25% in the tumor sample, less than 5% in the normal samples and identified by at least 5 independent reads, were considered for the subsequent analyses.

SM5. validation of SNV and mutations detected by exome sequencig

After exome sequencing, over 100 SNVs and somatic mutations were manually selected for validation by PCR. Genomic DNAs (10 ng) were used as template for PCR amplification in 50 µl reactions containing 1x Phusion HF Buffer (NEB), 200 µM dNTPs, 0.5 µM each forward and reverse primers and 1 unit of Phusion High-Fidelity DNA polymerase (NEB). Primers specific to genomic DNA sequence flanking individual SNVs were designed using Primer3 software [14]. Amplification reactions were performed under following conditions: 1x: 95°C – 1 min; 25x: 95°C – 30 sec, 60°C – 30 sec, 72°C – 30 sec; 1x: 72°C – 5 min. PCR products were purified using PCR purification kit (Zymo Research). DNA concentrations were measured using NanoDrop ND-1000 Spectrophotometer and fragment lengths were confirmed on 2% (w/v) agarose gels. DNA sequencing was performed using universal primer 5′-GTCAAACGACGGCCAGT-3′.

SM6. analysis of the TCGA COAD and READ datasets

We downloaded and combined the data from the COAD (colon adenocarcinoma) and READ (rectum adenocarcinoma) databases from the TCGA (https://tcga-data.nci.nih.gov/). We employed Level 3 methylation data of 398 Human Methylation 450K (HM450) and 278 Human Methylation 27K (HM27) arrays, corresponding to 420 colon primary adenocarcinomas, 163 rectal primary adenocarcinomas, one rectal recurrent adenocarcinoma, 75 normal colonic mucosa and 12 rectal mucosa samples. For samples analyzed in duplicate (TCGA-A6–2672-01, TCGA-A6- 5661–01, TCGA-A6–5665-01, TCGA-AG-3728–01 and TCGA-AG-A026–01), ß-values of the probes of interest were combined by averaging across replicates. *MGMT* methylation status was determined by probes cg12434587, cg12981137 and cg02941816, common to both HM450 and HM27 platforms. These probes interrogate three CpG sites located –239 bp, 128 bp and 248 bp from the *MGMT* transcriptional start site, respectively, within regions previously shown to associate with transcription of *MGMT* [15]. ß-values of these probes are below 0.2 in all colorectal normal samples analyzed, but exhibit a bimodal distribution in tumors (Figure S6). Tumors with an average ß-value in these three probes greater than 0.2 were considered as hypermethylated. Level 2 mutational analysis performed by exome sequencing was available for 386 CRC patients. Clinical information was available for all patients except for patient TCGA-F4–6857, whose exome sequencing results were however included in the COAD somatic mutations database. A summary table of the data employed in this work is available as supporting table S1. Tumors with more than 10 mutations/Mb, also referred to as hypermutated tumors [16], were considered to have a mutator phenotype due to defects in the MMR system.

SUPPLEMENTARY FIGURES AND TABLE

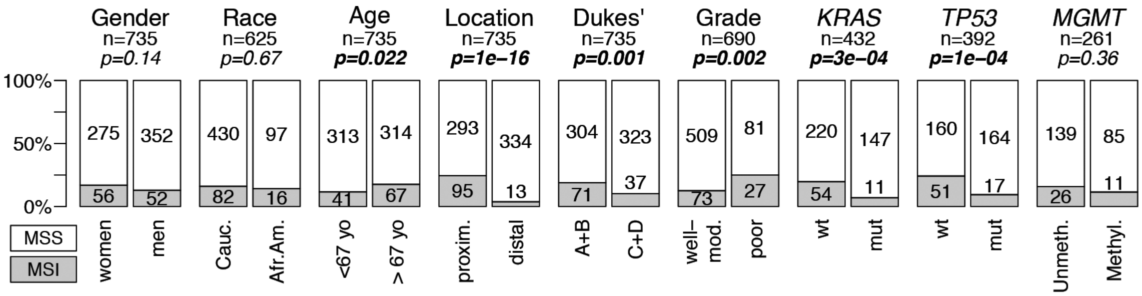

Supplementary Figure S1: Association of MSI phenotype with CRC patients phenotype and genotype. MSI tumors are in grey, and non-MSI tumors in white. MSI frequency was similar between Caucasians and African-Americans, and higher in females and older patients (Figure S1 top). There were more MSI tumors located proximal to the splenic flexure, at Dukes' stages A or B, and with a degree of poor differentiation (Figure 1s middle), and tumors with MSI had fewer mutated *KRAS* and *TP53* (Figure S1, bottom). These results are in agreement with our initial and subsequent studies on MSI in CRC (1, 9), *p*-values were obtained by Fisher's exact test. In bold type, *p*-values below 0.05.

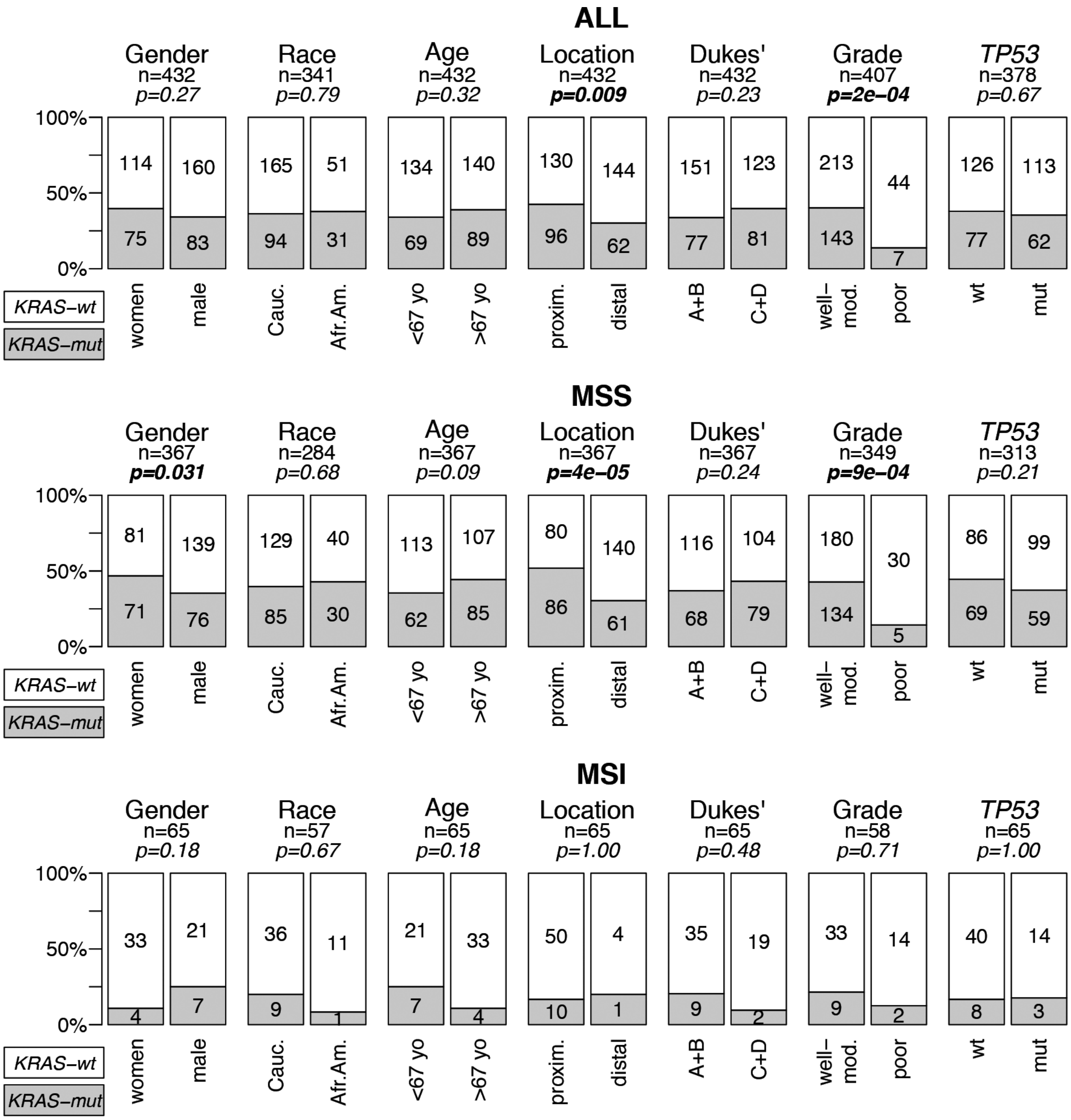

Supplementary Figure S2: Association of *KRAS* mutations with CRC patients phenotype and genotype in all (upper row), non-MSI (middle row) and MSI (lower row) CRC. In white, tumors with wild-type *KRAS* (*KRAS-wt*). In grey, tumors with mutations in codon 12 or 13 of *KRAS* (*KRAS-mut*). *p*-values were calculated by Fisher's exact test. Significant *p*-values are in bold.

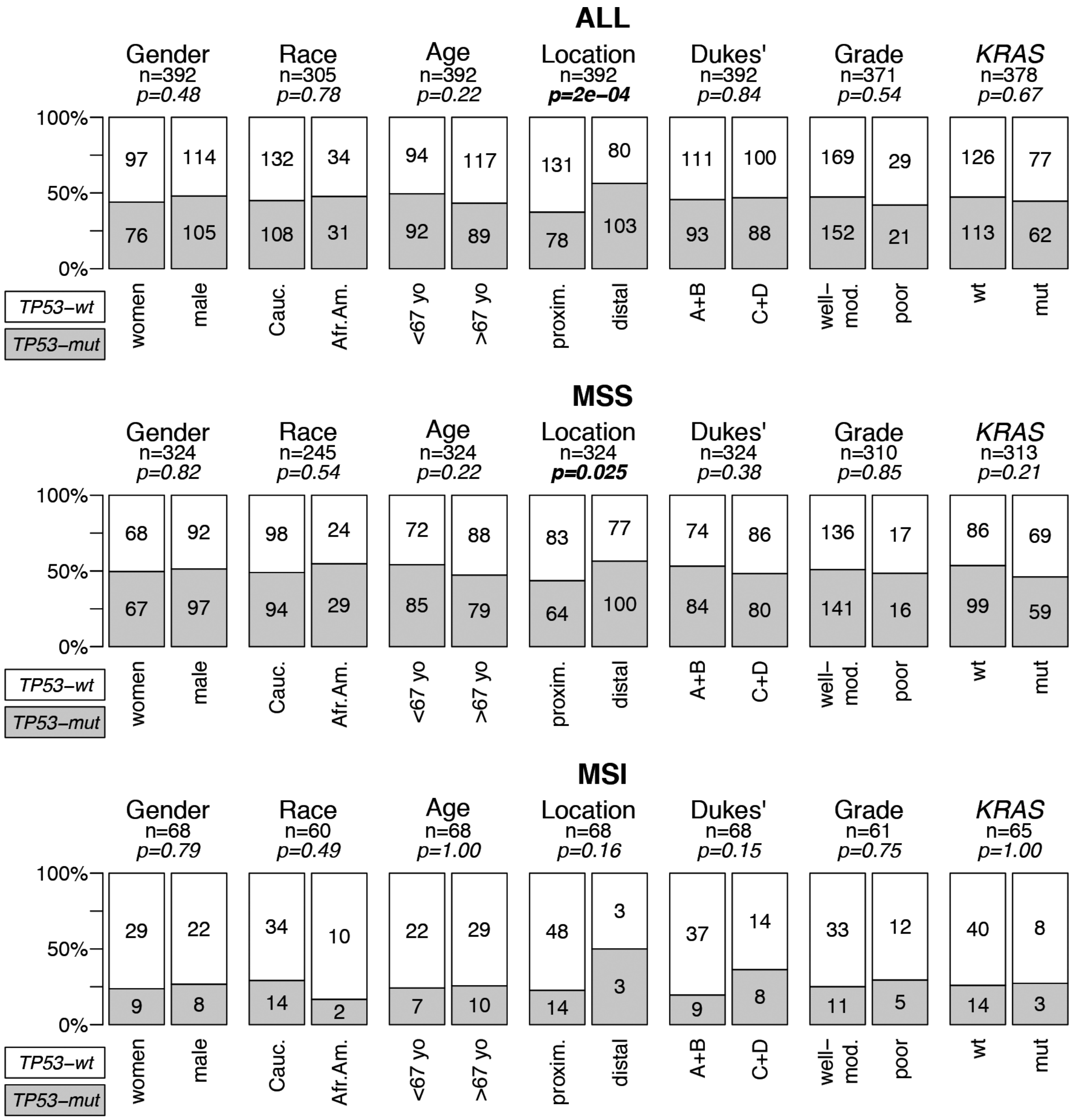

Supplementary Figure S3: Association of *TP53* mutations with CRC patients phenotype and genotype in all (upper row), non-MSI (middle row) and MSI (lower row) CRC. In white, tumors with wild-type *TP53* (*TP53-wt*). In grey, tumors with mutations in exons 5 to 9 of *TP53* (*TP53-mut*). *p*-values were calculated by Fisher's exact test. Significant *p*-values are in bold.

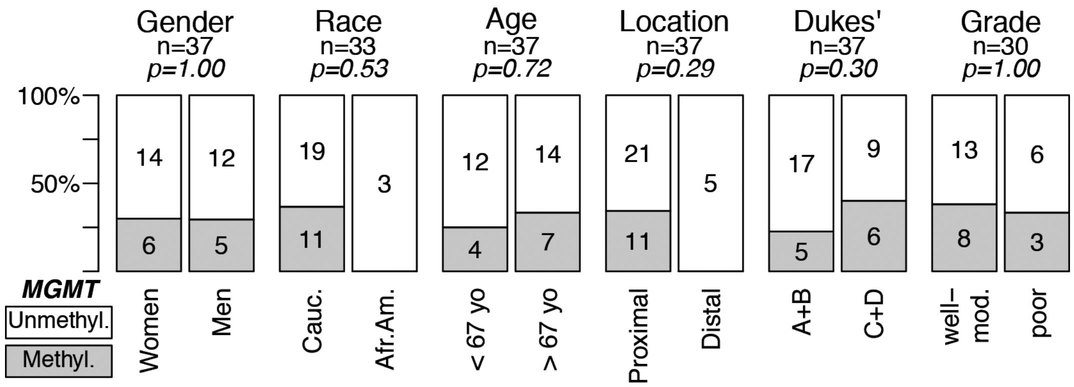

Supplementary Figure S4: Association of hypermethylation of *MGMT* promoter with phenotypic and genotypic characteristics in patients with MSI CRC. In grey *MGMT*-hypermethylated tumors, and in white tumors without hypermethylation. *MGMT* methylation was assessed by MSP (see materials and methods). *p*-values were calculated by univariate Fisher's exact test.

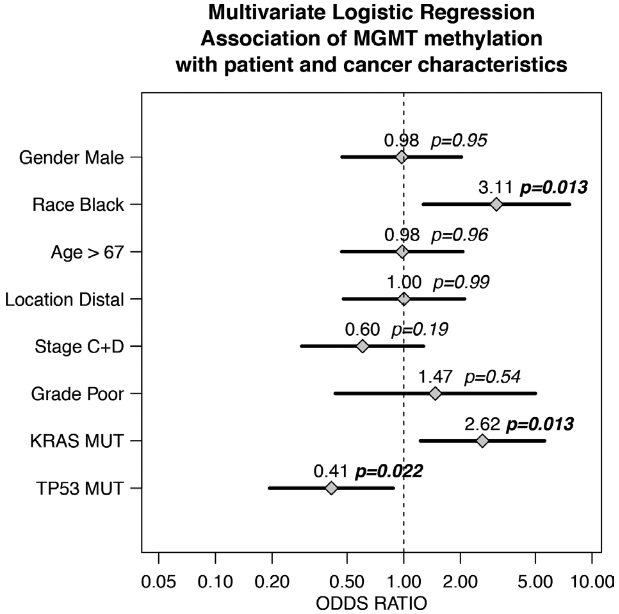

Supplementary Figure S5: Forest plot of multivariate logistic regression analysis of the association between *MGMT* methylation and the genotypic and phenotypic characteristics of the non MSI colorectal cancer patients in the study. Diamonds indicate the estimated odds ratios, and horizontal segments indicate the 95% confidence intervals. All two-factor interactions were non statistically significant and were discarded from the modeling.

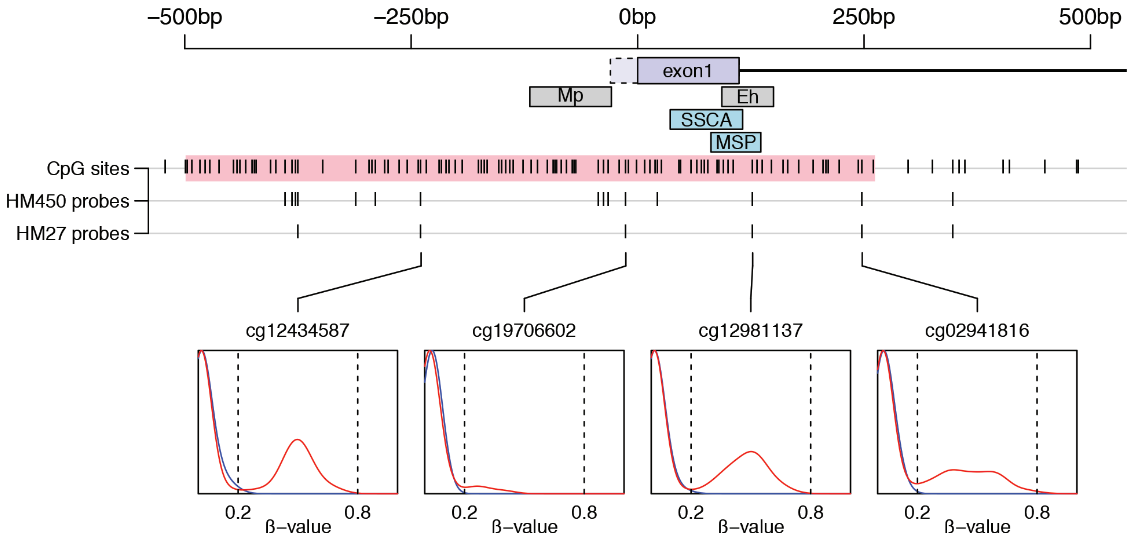

Supplementary Figure S6: Diagram of the 5' region of *MGMT* gene. Position 0 corresponds to the TSS annotated in the GRCh37 version of the human genome (Chromosome 10:131,265,448). In purple, the first exon, spawning 113 bp. A 30 bp longer version of this exon, appearing in several earlier publications, is indicated by a dashed box. Mp and Eh indicate the minimal promoter and the enhancer regions, respectively. The regions analyzed by MS-SSCA and MSP are indicated by the blue boxes. Both analyses overlap with the enhancer region, where hypermethylation exerts a dramatic effect on the transcriptional levels of *MGMT*. In pink, the 762 bp 5'-associated CpG island. The Illumina HM450K and HM27K probes mapping in this region are indicated. Six probes within this region are common to both platforms. The plots represent the density of the methylation values (ß-values) for the common probes closest to the TSS, in the normal (blue line) and tumor (red line) samples of the TCGA COAD+READ datasets. The ß-value of probes with a clear bimodal distribution (cg12434587, cg12981137 and cg02941816) was averaged, and those cases with a combined value higher than 0.2 were considered hypermethylated. The second peak of the bimodal distribution was centered around ß-value of 0.5, suggestive of monoallelic methylation.

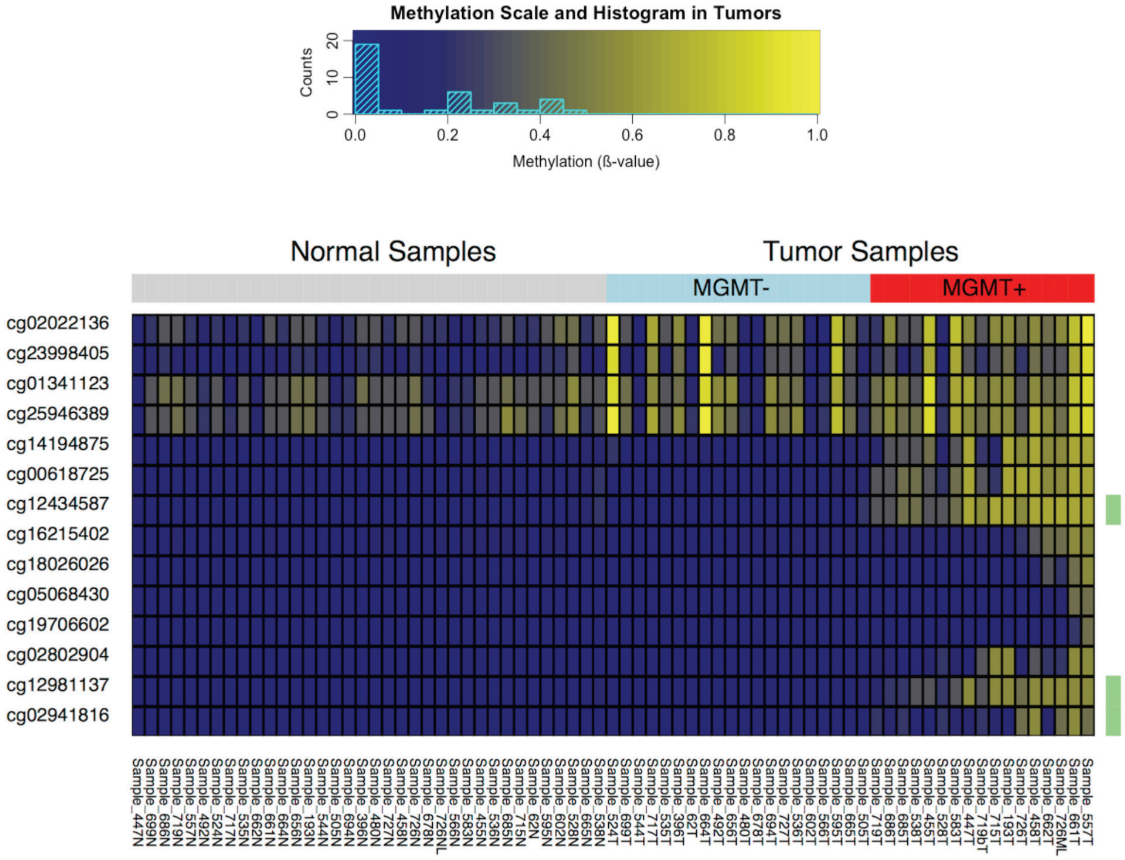

Supplementary Figure S7: Heatmap of the methylation at the 5' region of MGMT in normal and tumor samples from our CRC collection. Methylation was measured using Illumina HM450K arrays. In rows, probes at the 5' region of MGMT. In columns, the samples analyzed. Normal sample names end in "N" or "NL" (normal liver sample from patient 726). Tumor sample names end in "T" or "ML" (liver metastasis of patient 726). Green squares indicate the probes cg12434587, cg12981137 and cg02941816, which were averaged to classify the tumors into MGMT- (light blue) and MGMT+ (red). Average methylation of these probes exhibited a bimodal distribution (upper graph) with a second peak centered around ß-value 0.2~0.4, suggestive of monoallelic methylation and some degree of non-tumor tissue in the tumor samples.

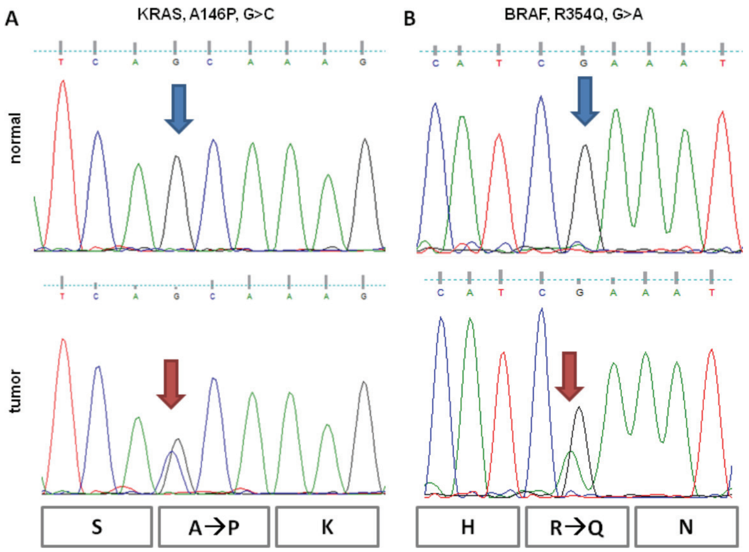

Supplementary Figure S8: Validation of somatic mutations in (A) *KRAS* (A146P, G > C) and (B) BRAF (R354Q, G > A) in CRC case 699 by Sanger sequencing. DNA sequencing trace electropherograms for matching normal and tumors samples are presented. Red arrows indicate mutated nucleotides in the tumor samples (bottom panels). Blue arrows indicate corresponding nucleotides in the matching normal samples (upper panels). Amino acid residues are shown in rectangles below.

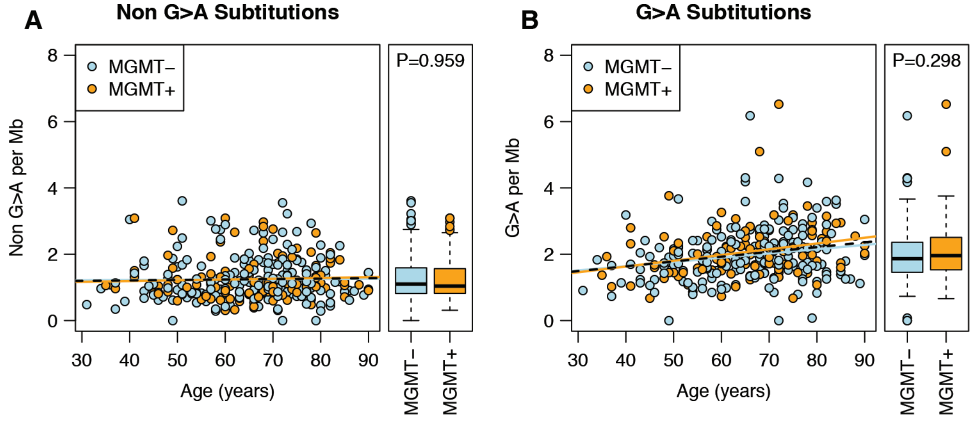


```
Multivariate Regression Analysis

        Coefficient        Prob
Age     0.0013±0.0031     0.665
MGMT    0.0086±0.0798     0.915
```

```
Multivariate Regression Analysis

        Coefficient        Prob
Age     0.0145±0.0037   1.2e-04 ***
MGMT    0.1058±0.0965     0.274
```

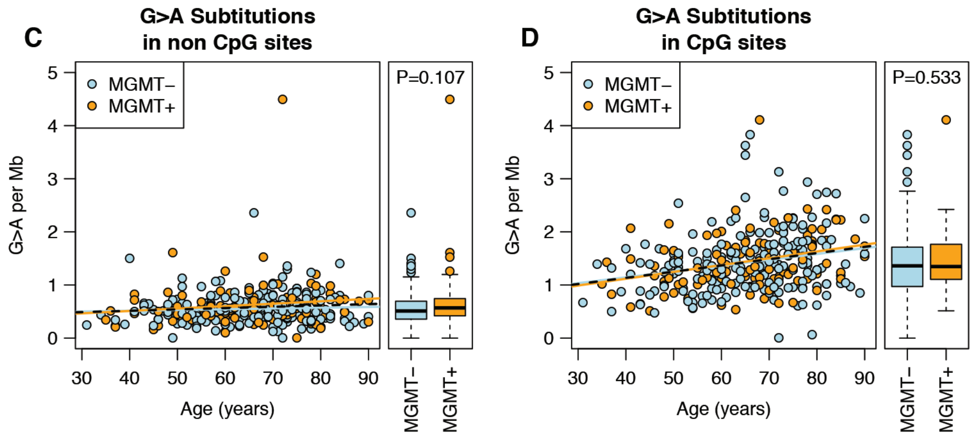


```
Multivariate Regression Analysis

        Coefficient        Prob
Age     0.0025±0.0017     0.131
MGMT    0.0756±0.0436     0.084 .
```

```
Multivariate Regression Analysis

        Coefficient        Prob
Age     0.0117±0.0026   1.0e-05 ***
MGMT    0.0207±0.0673     0.758
```

Supplementary Figure S9: Association of somatic single nucleotide mutations with patient age and *MGMT* methylation status. Association of non G > A (A), G > A (B), G > A in non-CpG sites (C) and G > A in CpG sites (D) mutations with patient age (scatterplots) or *MGMT* methylation status (boxplots, *p*-value calculated by Wilcox test). In orange, MGMT-methylated cases (MGMT+); in blue, MGMT-unmethylated cases (MGMT−). Below each scatterplot graph, we show the bivariate linear regression analysis considering both patient age and *MGMT* status as factors. Significance codes: $p < 0.1$ (.), $p < 0.05$ (*), $p < 0.01$ (**) and $p < 0.001$ (***). G > A substitutions associate with patient age (panel B, $p = 1.2 \times 10^{-4}$), especially those occurring at CpG sites (panel D, $p = 1.0 \times 10^{-5}$). When considering all somatic single nucleotide substitutions together (G > A plus non G > A, not shown) we found an association with age ($p = 0.009$) but not with *MGMT* methylation status ($p = 0.464$).

Supplementary Table S1: Primers and annealing Tm for *TP53* mutational analysis

Oligo	Sequence	T (ºC)
Exon 4F	5′-TTCACCCATCTACAGTCC-3′	62
Exon 4R	5′-TCAGGGCAACTGACCGT-3′	
Exon 5F	5′-TTCCTCTTCCTGCAGTACTCC-3′	62
Exon 5R	5′-GCCCCAGCTGCTCACCATCG-3′	
Exon 6F	5′-CACTGATTGCTCTTSGGTCT-3′	54
Exon 6R	5′-AGTTGCAAACCAGACCTCAGG-3′	
Exon 7F	5′-TCTCCTAGGTTGGCTCTGAC-3′	58
Exon 7R	5′-CAAGTGGCTCCTGACCTGGA-3′	
Exon 8F	5′-CCTATCCTGAGTAGTGGTAA-3′	54
Exon 8R	5′-CCTGCTTGCTTACCTCG-3′	

REFERENCES

1. Zauber NP, Wang C, Lee PS, Redondo TC, Bishop DT, Goel A. Ki-ras gene mutations, LOH of the APC and DCC genes, and microsatellite instability in primary colorectal carcinoma are not associated with micrometastases in pericolonic lymph nodes or with patients' survival. J Clin Pathol. 2004; 57:938–942.
2. Clark SJ, Harrison J, Paul CL, Frommer M. High sensitivity mapping of methylated cytosines. Nucleic Acids Res. 1994; 22:2990–2997.
3. Paulin R, Grigg GW, Davey MW, Piper AA. Urea improves efficiency of bisulphite-mediated sequencing of 5'-methylcytosine in genomic DNA. Nucleic Acids Res. 1998; 26:5009–5010.
4. Esteller M, Hamilton SR, Burger PC, Baylin SB, Herman JG. Inactivation of the DNA repair gene O6-methylguanine-DNA methyltransferase by promoter hypermethylation is a common event in primary human neoplasia. Cancer Res. 1999; 59:793–797.
5. Lind GE, Thorstensen L, Lovig T, Meling GI, Hamelin R, Rognum TO, Esteller M, Lothe RA. A CpG island hypermethylation profile of primary colorectal carcinomas and colon cancer cell lines. Mol Cancer. 2004; 3:28.
6. Assenov Y, Müller F, Lutsik P, Walte J, Lengauer T, Bock C. Comprehensive Analysis of DNA Methylation Data with RnBeads. Nature Methods. 2014; in press.
7. Lohse M, Bolger AM, Nagel A, Fernie AR, Lunn JE, Stitt M, Usadel B. RobiNA: a user-friendly, integrated software solution for RNA-Seq-based transcriptomics. Nucleic Acids Res. 2012; 40:W622–627.
8. Li H, Durbin R. Fast and accurate short read alignment with Burrows-Wheeler transform. Bioinformatics. 2009; 25:1754–1760.
9. Hummel M, Bonnin S, Lowy E, Roma G. TEQC: an R package for quality control in target capture experiments. Bioinformatics. 2011; 27:1316–1317.
10. McKenna A, Hanna M, Banks E, Sivachenko A, Cibulskis K, Kernytsky A, Garimella K, Altshuler D, Gabriel S, Daly M, DePristo MA. The Genome Analysis Toolkit: a MapReduce framework for analyzing next-generation DNA sequencing data. Genome Res. 2010; 20:1297–1303.
11. DePristo MA, Banks E, Poplin R, Garimella KV, Maguire JR, Hartl C, Philippakis AA, del Angel G, Rivas MA, Hanna M, McKenna A, Fennell TJ, et al. A framework for variation discovery and genotyping using next-generation DNA sequencing data. Nat Genet. 2011; 43:491–498.
12. Cingolani P, Platts A, Wang le L, Coon M, Nguyen T, Wang L, Land SJ, Lu X, Ruden DM. A program for annotating and predicting the effects of single nucleotide polymorphisms, SnpEff: SNPs in the genome of Drosophila melanogaster strain w1118, iso-2; iso-3. Fly (Austin). 2012; 6:80–92.
13. Koboldt DC, Chen K, Wylie T, Larson DE, McLellan MD, Mardis ER, Weinstock GM, Wilson RK, Ding L. VarScan: variant detection in massively parallel sequencing of individual and pooled samples. Bioinformatics. 2009; 25:2283–2285.
14. Rozen S, Skaletsky H. Primer3 on the WWW for general users and for biologist programmers. Methods Mol Biol. 2000; 132:365–386.
15. Nakagawachi T, Soejima H, Urano T, Zhao W, Higashimoto K, Satoh Y, Matsukura S, Kudo S, Kitajima Y, Harada H, Furukawa K, Matsuzaki H, et al. Silencing effect of CpG island hypermethylation and histone modifications on O6-methylguanine-DNA methyltransferase (MGMT) gene expression in human cancer. Oncogene. 2003; 22:8835–8844.
16. The Cancer Genome Atlas Research Network. Comprehensive molecular characterization of human colon and rectal cancer. Nature. 2012; 487:330–337.
